# Knowledge, Attitude, and Practice of Intranasal Corticosteroid in Allergic Rhinitis Patients: Development of a New Questionnaire

**DOI:** 10.3390/healthcare10010008

**Published:** 2021-12-22

**Authors:** Senthilraj Retinasekharan, Norasnieda Md Shukri, Ahmad Filza Ismail, Baharudin Abdullah

**Affiliations:** 1 Department of Otorhinolaryngology-Head & Neck Surgery, School of Medical Sciences, Universiti Sains Malaysia, Kubang Kerian 16150, Kelantan, Malaysia; drsenthilraj@hotmail.com (S.R.); asnieda@usm.my (N.M.S.); 2 Department of Community Medicine, School of Medical Sciences, Universiti Sains Malaysia, Kubang Kerian 16150, Kelantan, Malaysia; afilza@usm.my

**Keywords:** allergic rhinitis, intranasal corticosteroids, knowledge, attitude, practice

## Abstract

Objectives: The knowledge gap and attitude of allergic rhinitis (AR) patients using intranasal corticosteroid (INCS) led to the poor outcome of their disease. We aimed to develop and validate a new questionnaire to assess the knowledge, attitude, and practice (KAP) of AR patients that can be used to assess and understand the factors affecting compliance of INCS. Methods: The questionnaire comprised development and validation stages. A self-administered questionnaire was developed after a comprehensive literature review. It was subjected to content and face validity before a revised final version was drafted. Exploratory factor analysis was used to assess the validity of the questionnaire. Cronbach’s alpha was used to verify internal consistency. Results: The development phase resulted in a questionnaire consisting of 14 items. Explanatory factor analysis revealed four factors associated with KAP. The four factors were extracted, and 12 items were kept. The factors were attitude domain with four items (factor 1), practice domain with four items (factor 2), and knowledge domain with four items (factor 3 has two items, and factor 4 has two items). The Cronbach’s alpha of the four factors ranged from 0.614 to 0.809. The final questionnaire consists of 3 domains with 12 items (the knowledge domain with four questions; the attitude domain with four questions; the practice domain with four questions) and was valid and reliable. Conclusions: The newly developed questionnaire has adequate validity and reliability. It is a useful tool to improve the treatment of AR patients by understanding the factors affecting their compliance.

## 1. Introduction

Intranasal corticosteroid (INCS) is highly recommended for the treatment of allergic rhinitis (AR), which is the preferred agent of choice over oral H1-antihistamines, oral leukotriene receptor antagonists, and intranasal H1-antihistamines for patients with seasonal and persistent AR [1,2]. Consistent prophylactic use of INCS is effective in reducing rhinorrhea, nasal blockage, itching, and sneezing in both children and adults [3,4]. AR has been shown to have a significant negative impact on patients’ activities of daily living and their quality of life and affects their emotional well-being, productivity, and cognitive functioning [5]. Consequently, there is considerable economic burden that include direct and indirect costs caused by absenteeism and decreased productivity at school or work. A survey by Katelaris et al. [5] showed adherence to INCS and its regular use improves the quality of life of sufferers significantly. Based on the survey, 64% stated that most or all symptoms were effectively relieved, and only 4% reported no significant symptom relief. This established compliance to INCS is crucial in the management of AR.

Although INCS is the most prescribed AR treatment by doctors, less than half of patients are fully satisfied with their INCS. Some of the most common reasons for patients to discontinue treatment relate to lack of long-lasting symptom relief and perceived side effects [6]. Studies done on knowledge and attitude towards INCS among physicians and non-AR patients, studies describing the attitude and practices on AR among different socioeconomic classes, and studies on the physician’s opinion on the prevention and treatment of AR showed significant knowledge gap among attending physicians and patients [7,8,9]. Although the misperceptions about INCS have been identified and the occurrence of treatment gap acknowledged in patients, a strategy to resolve these issues has not been successful. This is partly due to the lack of understanding of the knowledge, attitude, and practice (KAP) of patients towards INCS. The knowledge gap and attitude of patients prescribed with INCS adversely affects the outcome of their condition. These shortcomings could be rectified by having a specific tool devoted to an assessment of self-reported evaluation of KAP. The aim of this study was to develop a validated questionnaire to assess the KAP of AR patients towards INCS.

## 2. Materials and Methods

### 2.1. Questionnaire Development

The questionnaire was developed after a comprehensive literature review. The preliminary version of the questionnaire, consisting of 16 items, was given to 8 researchers and experts in the field (7 otorhinolaryngologists and 1 public health physician) (Supplementary Figure S1). They were asked to comment on the context and content of the items. Each reviewer independently rated the relevance of each item on each domain of the questionnaire to the conceptual framework using a 4-point Likert scale (1 = not relevant, 2 = somewhat relevant, 3 = relevant, 4 = very relevant). The content was assessed by the Content Validity Index (CVI), which is the most widely used method for content validity in instrument development and computed using the Item-CVI (I-CVI) [10]. I-CVI is calculated as the number of experts’ ratings of “very relevant” for each item divided by the total number of experts, with values range from 0 to 1. When I-CVI > 0.79, the item is relevant; between 0.70 and 0.79, the item needs revisions; and if the value is below 0.70, the item is eliminated [10].

This questionnaire was further pre-tested with 20 AR patients at another hospital not involved in this study who were able to read and write in English. The participants were asked to answer and highlight ambiguous or problematic items by rating each items on a Likert scale of 1 to 4 (strongly disagree = 1, disagree = 2, agree = 3, and strongly agree = 4). It was to test the face validity of the questionnaire in order to assess how meaningful the concepts were to the studied community, the clarity of the wordings, and the likelihood the target audience would be able to answer the questions. The layout and appearance of the questions were modified based on the face validation. A revised draft of the KAP towards INCS (KAP-INCS) questionnaire consisting of 14 items was constructed according to the concepts measured by each of the three domains (Table 1).

The KAP-INCS questionnaire was divided into two sections, the demographic data and KAP towards INCS use. The demographic section consists of seven questions, such as age, gender, ethnicity, residency, education qualifications, the year of diagnosis, and the year nasal spray was prescribed. The second section was the assessment of the KAP towards INCS use. The knowledge domain consists of five questions, attitude domain consists of five questions, and the practice domain consists of four questions. The knowledge domain consists of 5 close-ended statements with three possible answers: “yes,” “no,” and “not sure.” The “yes” answer was given a score of two, the “no” answer was given a zero score, and “not sure” was given a score of one. The attitude domain consists of six ordered scores “totally disagree, disagree, quite disagree, quite agree, agree, and totally agree.” The practice domain consists of five ordered score: “almost never, rarely, sometimes, almost always, and always.” Likert-scale questions were used to collect data for all of the three domains.

### 2.2. Study Setting and Participants

The final version of KAP-INCS was given to patients at two tertiary hospitals (i.e., Hospital Pulau Pinang and Hospital Universiti Sains Malaysia) over a period of 9 months for self-administration. The selected patients were above 15 years old of age, who were able to read and write in English, previously diagnosed as AR, and currently being treated by INCS. Patients with self-diagnosed AR and on self-medicated nasal sprays were excluded from the study. Sample size was determined using factor analysis method with a subject-to-variable ratio of 1:5 [11]. The sample size obtained was 77. Consent was obtained, and anonymity of the participants was maintained. The study protocol was reviewed and approved by the Human Research Ethics Committee, Universiti Sains Malaysia (No: USM/JEPEM/17030153) and was performed in adherence with the Declaration of Helsinki.

### 2.3. Validation of Questionnaire

The exploratory factor analysis and Cronbach’s alpha were used to measure construct validity and internal consistency of the KAP-INCS questionnaire [12]. The factor analysis, Kaiser–Meyer–Olkin test (KMO), and Bartlett’s test of sphericity were computed to identify the items to be included in the final analysis. A typical factor analysis was performed based on Pearson correlations since the Likert scale could be treated as an interval or ratio scale. Principal axis factoring with rotation method of promax with Kaiser’s normalization and scree plot inspection was used to determine the number of factors to retain. According to Kaiser’s criterion, all factors with eigenvalues < 1 were dropped. Secondly, the factor analysis was repeated by including and excluding each item until the best combination or reduction was met. Lastly, the factor analysis was again computed to produce factor loading for the final version of the questionnaire. Factor loadings > 0.5 and communalities of >0.25 were considered acceptable. In general, correlations of <0.85 between factors are expectable in health sciences [13]. Once the validity procedures were completed, the final version of the KAP-INCS questionnaire was examined to assess its reliability. For internal consistency reliability, a Cronbach’s alpha coefficient > 0.65 was considered acceptable.

### 2.4. Statistical Analysis

Continuous variables were reported as the mean value ± standard deviation (SD). Bartlett’s test for sphericity was to test the appropriateness of the factor model, while the KMO measure of Sampling Adequacy was to test whether the partial correlations among variables were small. The KMO statistic ranged between 0 and 1 [14]. KMO value close to 1 indicates the sample efficiency and justifiability for factor analysis. From the Pearson’s correlation matrix, items that show weak correlation with others would be removed. Cronbach’s alpha coefficient was used as an estimate of the internal consistency of the questionnaire.

## 3. Results

Seventy-seven patients consisting of 39 males and 38 females enrolled in this study. The age ranged from 15 to 77 years, with a mean age of 36.74. Further details of the patients’ demography are shown in Table 2. 

### 3.1. Content Validity

Based on the comments of the experts, two items from the knowledge domain in the preliminary questionnaire were deleted, as they were ambiguous and did not serve to answer the objective of the present study. The two questions, “I know the symptoms of allergic rhinitis” (K-Q1) and “Allergic rhinitis can be prevented” (K-Q2) were deemed to test knowledge of the disease rather than the assessment of the INCS. Fourteen items remained, consisting of five items in knowledge domain, five items in attitude domain, and four items in practice domain (Supplementary Figure S2). One item on the draft of the 14 items questionnaire was deemed to be inappropriate because it yielded CVI of 0.5 (4/8) and was replaced. That item was from the knowledge domain: “I recognize the importance of using nasal steroid” (K-Q1) and was replaced by “I am aware of the importance of using nasal steroid,” which yielded CVI of 1.0 (8/8). All the remaining items were valid, with CVI ranging from 0.87 (7/8) to 1.0 (8/8), and were retained.

### 3.2. Face Validity

All 20 pretested participants rated each parameter at three or four on a Likert scale of 1 to 4. Ninety-five percent indicated they understood the questions and found them easy to answer, and 90% indicated the appearance and layout would be acceptable to the intended target group. The remaining items of the questionnaire that underwent statistical analysis along with their descriptive statistics are presented in Table 3, Table 4 and Table 5.

### 3.3. Construct Validity

KMO measure of sampling adequacy was 0.655 (>0.5) and Bartlett’s test of sphericity was appropriate. Thus, a satisfactory factor analysis could proceed. The exploratory factorial analysis showed four factors with eigenvalue of more than one. This was supported by a scree plot, which also indicated four factors. On the first run of exploratory factor analysis (EFA), question A-Q2 (“My knowledge of allergic rhinitis is sufficient”) was marked for deletion, as the communalities was 0.223 (<0.25). Next, the question K-Q1 (“I am aware of the importance of using nasal steroid”) was deleted, as it showed a factor loading of <0.5 and communalities < 0.25. Then, item extraction and another run of EFA were performed. All items showed communalities > 0.25. All had factor loading > 0.5 except for P-Q3 (0.35) and factor correlation coefficient < |0.85|. However, item P-Q3 (“I use other prescribed medication without fail”) was accepted because we deemed it as important to the relevant domain and as having significant clinical value in determining the practice of the patient. Four factors were extracted, and 12 items were kept. The factors were divided as factor 1 (A-Q1, A-Q3, A-Q4, and A-Q5); factor 2 (P-Q1, P-Q2, P-Q3, and P-Q4); factor 3 (K-Q2 and K-Q3); and factor 4 (K-Q4 and K-Q5). Factor correlation (*r*) ranged from 0.102 to 0.345. The knowledge domain was divided into two factors, with items K-Q2 and K-Q3 in one factor (factor 3) and items K-Q4 and K-Q5 in another factor (factor 4) as per the Kaiser’s eigenvalue > 1 rule and factors correlation < 0.85.

### 3.4. Internal Consistency

**Figure 1:**
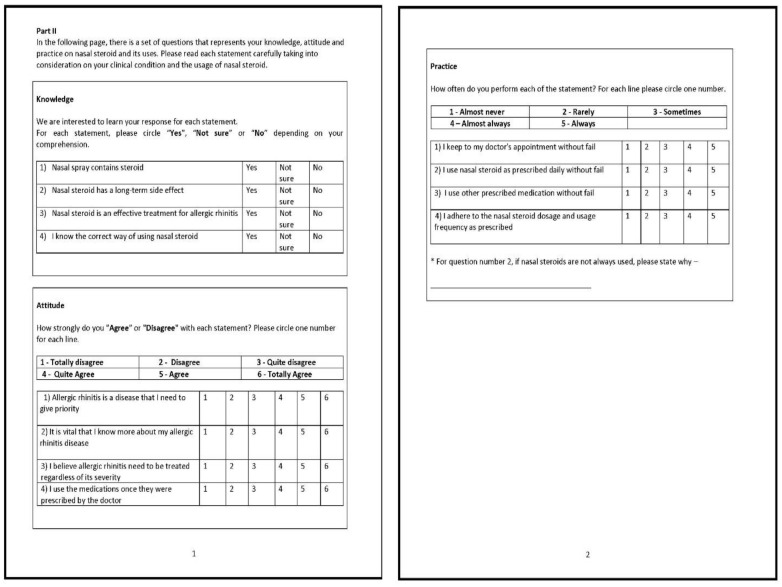
The final validated KAP-INCS questionnaire consists of 12 items (knowledge domain consists of four questions; attitude domain consists of four questions; and practice domain consists of four questions).

The Cronbach’s alpha was calculated for each factor. The Cronbach’s alpha for factor 1 was 0.809, factor 2 was 0.774, factor 3 was 0.735, and factor 4 was 0.614 (Table 6). Even though factor 4 was less than 0.65, for an exploratory research, it was considered to have marginally acceptable reliability [15], and factor 4 was kept in the questionnaire. The final validated questionnaire consists of three domains with 12 items; the knowledge domain consists of four questions, attitude domain consists of four questions, and the practice domain consists of four questions, as shown in Figure 1.

## 4. Discussion

The prevalence of AR is increasing worldwide, a trend that is connected with a variety of factors, such as changing global climate conditions, improvements in hygiene, changes in diet, and increased obesity [6]. Although INCS is proven to be efficacious for AR, their conditions are still not fully treated with the use of INCS. Poor knowledge and practice pattern among patients towards AR and the causative allergens could be the contributing factors [16]. There was poor awareness of AR among diagnosed and undiagnosed patients and the knowledge about the associated risk factors was found to be inadequate [17].

The present study provides an assessment on the validity and reliability of a newly developed KAP-INCS questionnaire to assess KAP of AR patients towards INCS use. Validation of this questionnaire, which includes content validity, face validity, construct validity, and reliability, is important because it aids physicians to understand the factors affecting compliance of INCS and allows them to improve the treatment of their AR patients. It is short and easily understood by patient but covers pertinent questions towards assessing their KAP. Content validity was determined after a review obtained from the experts in the field. The three domains consist of 16 questions initially, which was reduced to 14 questions after the content validation. The layout and appearances of the questions were modified after the face validation by pretesting with 20 AR patients. Finally, the three domains had 12 questions with four factors following construct validation, which showed an acceptable reliability.

Despite the availability of other pharmacological therapy for AR, INCS remains as one of its most effective treatment. It is superior to oral antihistamine in treating symptoms of AR and able to achieve sustained improvement of symptoms for the majority of patients [2]. Therefore, it is perplexing that patients still fail to get appropriate relief of their symptoms. One of the reasons is the different expectations between physician and patient in the treatment of AR. This disagreement eventually leads to major unmet need in their treatment. The major barrier in mending the discrepancy of patient and physician expectation is the lack of a specific tool for their evaluation. A self-reported questionnaire is a good instrument for the assessment of each specific domain of patient’s KAP. It allows the treatment of each patient to be individualized and customized to their needs and preferences, avoiding the “one size fits all approach,” which does not take into account individual patient requirement. When they are recognized, their doubts and concerns could be addressed with proper counselling.

An important feature of any patient care should consider each individual attitudes and beliefs responsible for their compliance to therapy. The perceived benefits and barriers in such an approach play a vital role in achieving therapeutic success. The perceptions, beliefs, and preferences of patients with AR may be barriers to starting and adhering to INCS therapy. The potential benefits of KAP-INCS questionnaire when implemented in clinical practice can be illustrated as follows. Fear of side effects has been reported frequently for INCS among patients, with the most common specific fears being damage to mucous membranes and organ-specific damage [18]. Although fear of side effects is well established, in real-life clinical practice, this has not been appraised adequately, resulting in poor compliance. With the KAP-INCS questionnaire, usage (P-Q2) and adherence of prescribed INCS (P-Q4) may be assessed, and non-adherence and poor compliance readily identified. The root cause of this poor practice may be recognized from K-Q2, which is “fear about the long term side effect of ICNS,” to allow immediate intervention. Another issue with the use of INCS is the sensory perceptions and patient preferences for selected INCS [19,20,21]. Sensory attributes of an INCS, including the scent, both the taste and aftertaste, drip down throat, running out from nose, and pain, affect patient adherence to its use. It can be predict when the usage (P-Q2) and adherence of prescribed INCS (P-Q4) show non-compliance without any identifiable contributing factors from both attitude (A-(Q1-4)) and knowledge (P-(Q1-4)) domains. This should raise suspicion towards issues with the specific characteristic of the INCS and allow the specific preference of each patient to be personalized.

## 5. Conclusions

The newly developed questionnaire is a valid and reliable tool to measure KAP among AR patients towards INCS. Understanding their KAP facilitates health-care providers to target patients and problem areas that need interventions.

## Figures and Tables

**Table 1:** The generation of question components for the construct of knowledge, attitude, and practice questionnaire.

Components	Item	Concepts Measured	Response Options
Knowledge (actual information from training or experience)	5 questions	To gauge knowledge of INCS	Yes; No; Not sure
Attitude (a settled way of thinking or feeling about something)	5 questions	To assess general attitude, behaviour, and cognitive factors towards INCS	Totally disagree; Disagree; Quite disagree; Quite agree; Agree; Totally agree
Practice (actual application of an idea, belief, or method)	4 questions	To evaluate common practice of INCS	Almost never; Rarely; Sometimes; Almost always; Always

INCS, intranasal corticosteroid.

**Table 2:** Socio-demographic characteristic of patients.

	*n* (%)
**Gender**	
Male	38 (49.4)
Female	39 (50.6)
**Ethnicity**	
Malay	29 (37.7)
Chinese	26 (33.8)
Indian	18 (23.4)
Others	4 (5.2)
**Education**	
Phd/Masters	2 (2.6)
Bachelor degree	42 (54.5)
Diploma	9 (11.7)
Secondary	24 (31.2)
**Diagnosis**	
Mild intermittent	20 (26)
Mild persistent	32 (41.6)
Moderate severe intermittent	0 (0)
Moderate severe persistent	25 (32.5)

**Table 3:** Descriptive statistics of the items in the knowledge domain.

Scale	Items	Mean (SD)	Yes (*n* %)	Not Sure (*n* %)	No (*n* %)
K-Q1	I am aware of the importance of using nasal steroid	1.62 (0.69)	57 (74.0)	11 (14.3)	9 (11.7)
K-Q2	Nasal spray contains steroid	1.49 (0.64)	44 (57.1)	27 (35.1)	6 (7.8)
K-Q3	Nasal steroid has a long-term side effect	1.39 (0.71)	40 (51.9)	27 (35.1)	10 (13.0)
K-Q4	Nasal steroid is an effective treatment for allergic rhinitis	1.61 (0.59)	51 (66.2)	22 (28.6)	4 (5.2)
K-Q5	I know the correct method of using the nasal steroid	1.65 (0.58)	54 (70.1)	19 (24.7)	4 (5.2)

**Table 4:** Descriptive statistics of the items in the attitude domain.

Scale	Items	Mean (SD)	Totally Disagree (%)	Disagree (%)	Quite Disagree (%)	Quite Agree (%)	Agree (%)	Totally Agree (%)
A-Q1	Allergic rhinitis is a disease that I need to make a priority	5.09 (1.03)	2 (2.6)	0 (0)	2 (2.6)	11 (14.3)	32 (41.6)	30 (39.0)
A-Q2	My knowledge of allergic rhinitis is sufficient	4.27 (0.93)	1 (1.3)	2 (2.6)	9 (41.6)	32 (41.6)	29 (37.7)	4 (5.2)
A-Q3	It is vital that I know more about my allergic rhinitis disease	5.08 (1.20)	3 (3.9)	0 (0)	4 (5.2)	10 (13.0)	24 (31.2)	36 (46.8)
A-Q4	I believe allergic rhinitis need to be treated regardless of its severity	5.27 (0.93)	1 (1.3)	1 (13)	1 (1.3)	6 (7.8)	32 (41.6)	32 (41.6)
A-Q5	I use the medications once they are prescribed by the doctor	5.06 (1.03)	2 (2.6)	0 (0)	3 (3.9)	9 (11.7)	35 (45.5)	28 (36.4)

**Table 5:** Descriptive statistics of the items in the practice domain.

Scale	Items	Mean (SD)	Almost Never (%)	Rarely (%)	Sometimes (%)	Almost Always (%)	Always (%)
P-Q1	I keep my doctor’s appointment without fail	4.03 (1.16)	6 (7.8)	2 (2.6)	8 (10.4)	29 (37.7)	32 (41.6)
P-Q2	I use nasal steroid as prescribed daily without fail	3.73 (0.87)	1 (1.3)	4 (5.2)	24 (31.2)	34 (44.2)	14 (18.2)
P-Q3	I use other prescribed medication without fail	3.49 (1.11)	6 (7.8)	6 (7.8)	22 (28.6)	30 (39)	13 (16.9)
P-Q4	I adhere to the nasal steroid dosage and usage frequency as prescribed	3.9 (1.04)	3 (3.9)	4 (5.2)	16 (20.8)	30 (39)	24 (31.2)

**Table 6:** Construct validity and reliability.

Factor	Item	Factor Loading ^a^	Communality ^b^	Cronbach’s Alpha ^c^
1. Attitude	A-Q1	0.719	0.563	0.809
	A-Q3	0.667	0.445	
	A-Q4	0.845	0.674	
	A-Q5	0.705	0.532	
2. Practice	P-Q1	0.675	0.462	0.774
	P-Q2	0.824	0.634	
	P-Q3	0.513	0.497	
	P-Q4	0.717	0.564	
3. Knowledge	K-Q2	0.690	0.467	0.735
	K-Q3	0.866	0.775	
4. Knowledge 2	K-Q4	0.527	0.478	0.614
	K-Q5	0.856	0.700	

^a^ Factor loadings > 0.5 and ^b^ communalities of >0.25 are considered acceptable. ^c^ Cronbach’s alpha coefficient > 0.65 is considered acceptable.

## Data Availability

All data generated and analyzed during this study are included in this published article.

## Supplementary Materials

The following are available online at https://www.mdpi.com/article/10.3390/healthcare10010008/s1, Figure S1:The preliminary version of the KAP-INCS questionnaire consists of 16 items (knowledge domain consists of seven questions; attitude domain consists of five questions and practice domain consists of four questions). Figure S2: The draft KAP-INCS questionnaire following expert evaluation consists of 14 items (knowledge domain consists of five questions; attitude domain consists of five questions and practice domain consists of four questions).
