# Supplementary material for: Knowledge, Attitude, and Practice of Intranasal Corticosteroid in Allergic Rhinitis Patients: Development of a New Questionnaire
Source: Healthcare (Basel). 2021 Dec 22;10(1):8. doi: 10.3390/healthcare10010008 (PMC8775375; doi:10.3390/healthcare10010008)
Supplement: Supplementary file 1 [file healthcare-10-00008-s001.zip › healthcare-1484861-supplementary.pdf]

Part II

In the following page, there is a set of questions that represents your knowledge, attitude and practice on nasal steroid and its uses. Please read each statement carefully taking into consideration on your clinical condition and the usage of nasal steroid.

Knowledge

We are interested to learn your response for each statement.
For each statement, please circle "**Yes**", "**Not sure**" or "**No**" depending on your comprehension.

1)I know the symptoms of allergic rhinitis	Yes	Not sure	No
2) Allergic rhinitis can be prevented	Yes	Not sure	No
3)I recognize the importance of using nasal steroid	Yes	Not sure	No
4) Nasal spray contains steroid	Yes	Not sure	No
5) Nasal steroid has a long-term side effect	Yes	Not sure	No
6) Nasal steroid is an effective treatment for allergic rhinitis	Yes	Not sure	No
7) I know the correct way of using nasal steroid	Yes	Not sure	No

Attitude

How strongly do you "**Agree**" or "**Disagree**" with each statement? Please circle one number for each line.

**1 - Totally disagree**	**2 - Disagree**	**3 - Quite disagree**
**4 - Quite Agree**	**5 - Agree**	**6 - Totally Agree**

1) Allergic rhinitis is a disease that I need to give priority	1	2	3	4	5	6
2) My knowledge of allergic rhinitis is sufficient	1	2	3	4	5	6
3) It is vital that I know more about my allergic rhinitis disease	1	2	3	4	5	6
4) I believe allergic rhinitis need to be treated regardless of its severity	1	2	3	4	5	6
5) I use the medications once they were prescribed by the doctor	1	2	3	4	5	6

Practice

How often do you perform each of the statement? For each line please circle one number.

**1 - Almost never**	**2 - Rarely**	**3 - Sometimes**
**4 – Almost always**	**5 - Always**	

1) I keep to my doctor's appointment without fail	1	2	3	4	5
2) I use nasal steroid as prescribed daily without fail	1	2	3	4	5
3) I use other prescribed medication without fail	1	2	3	4	5
4) I adhere to the nasal steroid dosage and usage frequency as prescribed	1	2	3	4	5

* For question number 2, if nasal steroids are not always used, please state why – ____

Supplementary Figure S1. The preliminary version of the KAP-INCS questionnaire consists of 16 items (knowledge domain consists of seven questions; attitude domain consists of five questions and practice domain consists of four questions).

Part II

In the following page, there is a set of questions that represents your knowledge, attitude and practice on nasal steroid and its uses. Please read each statement carefully taking into consideration on your clinical condition and the usage of nasal steroid.

Knowledge

We are interested to learn your response for each statement.
For each statement, please circle "**Yes**", "**Not sure**" or "**No**" depending on your comprehension.

1) I am aware of the importance of using nasal steroid	Yes	Not sure	No
2) Nasal spray contains steroid	Yes	Not sure	No
3) Nasal steroid has a long-term side effect	Yes	Not sure	No
4) Nasal steroid is an effective treatment for allergic rhinitis	Yes	Not sure	No
5) I know the correct way of using nasal steroid	Yes	Not sure	No

Attitude

How strongly do you "**Agree**" or "**Disagree**" with each statement? Please circle one number for each line.

**1 - Totally disagree**	**2 - Disagree**	**3 - Quite disagree**
**4 - Quite Agree**	**5 - Agree**	**6 - Totally Agree**

1) Allergic rhinitis is a disease that I need to give priority	1	2	3	4	5	6
2) My knowledge of allergic rhinitis is sufficient	1	2	3	4	5	6
3) It is vital that I know more about my allergic rhinitis disease	1	2	3	4	5	6
4) I believe allergic rhinitis need to be treated regardless of its severity	1	2	3	4	5	6
5) I use the medications once they were prescribed by the doctor	1	2	3	4	5	6

Practice

How often do you perform each of the statement? For each line please circle one number.

**1 - Almost never**	**2 - Rarely**	**3 - Sometimes**
**4 – Almost always**	**5 - Always**	

1) I keep to my doctor's appointment without fail	1	2	3	4	5
2) I use nasal steroid as prescribed daily without fail	1	2	3	4	5
3) I use other prescribed medication without fail	1	2	3	4	5
4) I adhere to the nasal steroid dosage and usage frequency as prescribed	1	2	3	4	5

* For question number 2, if nasal steroids are not always used, please state why –
____

Supplementary Figure S2. The draft KAP-INCS questionnaire following expert evaluation consists of 14 items (knowledge domain consists of five questions; attitude domain consists of five questions and practice domain consists of four questions).
